# Prevalence and Risk Factors of Major Depressive Disorder Among Women at Public Antenatal Clinics From Refugee, Conflict-Affected, and Australian-Born Backgrounds

**DOI:** 10.1001/jamanetworkopen.2019.3442

**Published:** 2019-05-03

**Authors:** Susan J. Rees, Jane R. Fisher, Zachary Steel, Mohammed Mohsin, Nawal Nadar, Batool Moussa, Fatima Hassoun, Mariam Yousif, Yalini Krishna, Batoul Khalil, Jok Mugo, Alvin Kuowei Tay, Louis Klein, Derrick Silove

**Affiliations:** 1 Psychiatry Research and Teaching Unit, School of Psychiatry, Faculty of Medicine, University of New South Wales, Kensington, Australia; 2 The Jean Hailes Research Unit, School of Public Health and Preventive Medicine, Monash University, Melbourne, Australia

## Abstract

**Importance:**

Pregnancy may increase the risk of depression among women who self-identify as refugees and have resettled in high-income countries. To our knowledge, no large systematic studies among women with refugee backgrounds in the antenatal period have been conducted.

**Objectives:**

To compare the prevalence of major depressive disorder (MDD), trauma exposure, and other psychosocial risk factors among women who identify as refugees, women from the same conflict-affected countries, and women from the host nation and to test whether self-identification as a refugee indicates greater likelihood of prevalence and risk.

**Design, Setting, and Participants:**

This cross-sectional study was undertaken in 3 public antenatal clinics in Sydney and Melbourne, Australia, between January 2015 and December 2016. Overall, 1335 women (685 consecutively enrolled from conflict-affected backgrounds and 650 randomly selected from the host nation) participated. Data analysis was undertaken between June and September 2018.

**Exposures:**

One-hour interviews covering mental health, intimate partner violence, and other social measures.

**Main Outcome and Measures:**

World Health Organization measure for intimate partner violence and the Mini-International Neuropsychiatric Interview from the *Diagnostic and Statistical Manual of Mental Disorders* (Fourth Edition) for MDD. To make a diagnosis, 1 of 2 items relating to being consistently depressed for 2 weeks and 3 further symptoms that cause personal distress or psychosocial dysfunction were endorsed.

**Results:**

Overall, 1335 women (84.8% overall response rate), comprising 685 (51.3%) from conflict-affected countries (women self-identifying as refugees: 289 [42.2%]) and 650 (48.7%) from the host nation, participated. The mean (SD) age was 29.7 (5.4) years among women from conflict-affected backgrounds and 29.0 (5.5) years among women born in the host nation. Conflict-affected countries included Iraq (260 [38.0%]), Lebanon (125 [18.2%]), Sri Lanka (71 [10.4%]), and Sudan (66 [9.6%]). Women who identified as refugees reported higher exposure to 2 to 3 (67 [23.2%]) and 4 or more (19 [6.6%]) general traumatic events compared with women from the host nation (103 [15.8%] and 21 [3.2%], respectively). Women who identified as refugees also reported higher exposure to 1 (147 [50.9%]) and 2 or more (97 [33.6%]) refugee-related traumatic events compared with women from the host nation (86 [13.2%] and 20 [3.1%], respectively). Women who identified as refugees reported higher rates of psychological intimate partner violence than women born in the host nation (124 [42.9%] vs 133 [20.5%]; *P* < .001). Women who identified as refugees were less likely to identify 5 or more supportive family or friends compared with women born in the host nation (36 [12.5%] vs 297 [45.7%]; *P* < .001). A greater proportion of women who identified as refugees reported experiencing 3 or more financial stressors compared with women born in the host nation (65 [22.5%] vs 41 [6.3%]; *P* < .001). Women who identified as refugees had the highest prevalence of MDD (94 [32.5%]), followed by women from other conflict-affected backgrounds (78 [19.7%]), and women born in the host nation (94 [14.5%]).

**Conclusion and Relevance:**

Women identifying as refugees reported a higher prevalence of MDD and all the indicators of adversity related to that disorder. Even after risk factors were accounted for, refugee status was associated with risk of MDD. Assessing whether women attending an antenatal clinic self-identify as refugees may offer an important indicator of risk of MDD and a range of associated psychosocial adversities.

## Key Points

**Question:**

What is the prevalence and which risk factors are associated with major depressive disorder in women from conflict-affected backgrounds resettling in a high-income country, and does self-identification as a refugee signal a higher risk?

**Findings:**

In this cross-sectional study of 1335 women (685 recruited from conflict-affected backgrounds and 650 from the host nation), the prevalence of major depressive disorder was 14.5% for women born in the host nation compared with 19.7% for women from conflict-affected backgrounds and 32.5% for women who identified as refugees.

**Meaning:**

General and refugee-related traumatic events, intimate partner violence, low social support, and financial stress should be prioritized in resettlement policies and clinical settings to reduce major depressive disorder in women from conflict-affected backgrounds and those who identify as refugees.

## Teaser

This cross-sectional study compares the prevalence of major depressive disorder, trauma exposure, and other psychosocial risk factors among women who identify as refugees, women from the same conflict-affected countries, and women born in the host nation, Australia.

## Introduction

A quarter of the world’s population, or approximately 2 billion persons, have been exposed to mass conflict.^1^ Women in the reproductive period of life—a demographic group at heightened risk of depression—make up a significant portion of the population from conflict-affected countries who resettle in high-income countries, such as Australia.^2^ To our knowledge, there are no systematic studies focusing on depression and associated psychosocial risk factors among this subpopulation of women.^3^

Even in well-resourced, high-income countries such as Australia, there are few services providing culturally sensitive psychosocial and mental health interventions for women from conflict-affected countries. To ensure the most efficient use of these services, it is important to offer screening procedures to identify the women in greatest need.^1^ The antenatal setting may assist the process of identification given that these facilities are a natural point of convergence for women at risk of depression originating from conflict-affected countries.

In general, populations exposed to mass conflict and/or displacement are at heightened risk of common mental disorders, such as major depressive disorder (MDD).^4^ Major depressive disorder provides an important focus for research, given its high prevalence and the substantial levels of disability associated with the disorder.^5^ As indicated, pregnant women have a higher prevalence of MDD, and this may be particularly true of those migrating from conflict-affected countries, given their exposure to trauma and related psychosocial difficulties.^2,3^ Nevertheless, within the population of women from conflict-affected countries, there is likely to be substantial variation in exposure to these forms of adversity. An added complexity is that women from conflict-affected countries enter Australia (and other resettlement countries) under a range of immigration programs. In Australia, the programs include categories of general migrant, humanitarian, family reunion, special women at risk, and others, which makes formal visa status an inaccurate indicator of psychosocial risk. Therefore, an important question for screening at antenatal clinics is whether women self-identifying as refugees are at a heightened risk of having experienced various forms of trauma, ongoing psychosocial difficulties, and MDD.

Women who identify as refugees may be at heightened risk of MDD for several reasons.^4^ The refugee experience exposes women first to the traumatic events (TEs) of mass conflict and then to more general TEs (eg, accidents, natural disasters, incidental forms of violence and abuse) during the often prolonged period of transition as they pursue hazardous journeys and insecure places of temporary refuge on their route to countries of permanent resettlement.^6^ Little is known about the prevalence of intimate partner violence (IPV) within refugee families after arrival in resettlement countries, but it is possible that past exposure to trauma makes these women at increased risk of future IPV.^7,8^ Moreover, for women who identify as refugees, loss of family and social networks may add to their isolation and cultural alienation in the new country, thereby increasing risk of MDD, especially during pregnancy.^3,9^ Finally, material losses and difficulties finding work after settlement may result in more severe financial hardships for women who identify as refugees, another known risk factor of MDD.^10,11^ In summary, although experiences such as TE exposure, IPV, low levels of social support, and financial difficulties are likely to be universal in their association with MDD risk across pregnant women from a range of backgrounds, women who identify as refugees may be more exposed to these forms of adversity and may be more susceptible to their negative psychological effects. As a consequence, these women may manifest higher rates of MDD compared with other women from conflict-affected countries and women from the host country.

The broad objective of the present study was to identify the psychosocial risk factors and the prevalence of MDD among women from conflict-affected countries attending antenatal clinics in Australia. We sought to test whether self-identification as a refugee indicated that those from within a broader group of women from conflict-affected backgrounds are at heightened risk of past trauma and ongoing psychosocial adversity as well as MDD.

## Methods

### Participants and Recruitment

Participants were recruited between January 2015 and March 2016 to the Women Aware Together With Their Children (WATCH) study. The study was conducted at 3 public antenatal clinics located in refugee-dense geographic areas in Sydney and Melbourne, Australia. Consecutive women were recruited from Arabic-speaking countries, Sudan, and Sri Lanka (Tamil-speaking). These nations represented the largest intake groups from conflict-affected regions entering Australia and other high-income countries at the time of this study. By limiting the study to these language groups, we sought to contain both the problems of transcultural measurement error and small cell sizes. Country of origin was identified by clinic records, requests for an interpreter, or culturally recognizable surnames, and country of birth data were checked against clinic appointment lists. Recruitment occurred at a woman’s first appointment at the clinic, which most commonly occurred between 12 and 20 weeks’ gestation (range, 9-42 weeks). Women with overt psychosis, severe medical illness, and obvious intellectual impairment were excluded.

Women born in Australia attended the clinics in substantially larger numbers than those from conflict-affected countries. To undertake a parallel sampling strategy over a similar time frame, we applied a computer-generated selection procedure to identify a random subset of women from the host country on a daily basis. Women members of the research team who spoke the same language as eligible women approached them in the waiting room and, following consent, conducted interviews lasting a maximum of 1 hour in private areas of the clinic, with breaks for refreshments or to attend to children.

### Ethics and Research Personnel

The study was approved by the Southwestern Sydney Local Health District Human Research Ethics Committee. Participants provided written informed consent and were remunerated for their time. In total, 8 women field workers from appropriate language backgrounds were given extensive training, consisting of 3 formal training days followed by tests of competence. Training covered IPV, research methods and practice, sensitive interviewing techniques, and the use of the diagnostic and World Health Organization measures. Staff received ongoing support, monitoring, and supervision throughout the study. Interrater reliability tests were conducted serially to maintain standards, based on group observations of videotaped interviews. We adhered strictly to World Health Organization guidelines for conducting safe and ethical IPV research. This study followed Strengthening the Reporting of Observational Studies in Epidemiology (STROBE) reporting guideline.

### Survey Measures

#### Cultural Accuracy

All measures were selected based on their previous psychometric evaluations and use across cultures. Measures were subjected to rigorous assessment of cultural and linguistic accuracy in the languages used.^12^ After standard translation and back-translation procedures were performed, final refinements were made by groups of linguistic experts.

#### Sociodemographic Characteristics

We drew on the Australian National Census for items recording age, marital status, level of education and qualifications, household composition, employment, and housing status. These socially derived items can be benchmarked against the Australian population.

#### Mental Health Measures

We used the Mini-International Neuropsychiatric Interview (MINI) based on the *Diagnostic and Statistical Manual of Mental Disorders* (Fourth Edition) (*DSM-IV*) to assess current MDD.^13,14^ We selected *DSM-IV* in preference to *DSM-5* because the latter had not yet been used extensively across cultures at the commencement of the study.

The interview consisted of 9 items: (1) “Have you been consistently depressed or down, most of the day, nearly every day, for the past 2 weeks?”; (2) “In the past 2 weeks, have you been much less interested in most things or much less able to enjoy the things you used to enjoy most of the time?”; (3) “Was your appetite decreased or increased nearly every day? Did your weight decrease or increase without trying intentionally (ie, by ±5% of body weight or ±8 lbs or ±3.5 kgs, for a 160 lb/70 kg person in a month)?”; (4) “Did you have trouble sleeping nearly every night (difficulty falling asleep, waking up in the middle of the night, early morning wakening, or sleeping excessively)?”; (5) “Did you talk or move more slowly than normal or were you fidgety, restless, or having trouble sitting still almost every day?”; (6) “Did you feel tired or without energy almost every day?”; (7) “Did you feel worthless or guilty almost every day?”; (8) “Did you have difficulty concentrating or making decisions almost every day?”; and (9) “Did you repeatedly consider hurting yourself, feel suicidal, or wish that you were dead?” Women who answered yes for either item 1 or 2 and answered yes for at least 3 other items were classified as having MMD.

#### Traumatic Events

We assessed lifetime exposure to TEs based on the inventory used in the World Mental Health Survey.^6^ Overall, 13 items were classified as general TEs that could occur in any society: (1) “Were you ever kidnapped or held captive?”; (2) “Were you ever involved in a life-threatening automobile accident?”; (3) “Did you ever have any other life-threatening accident, including on your job?”; (4) “Did you ever have a life-threatening illness?”; (5) “As a child, were you ever badly beaten up by your parents or the people who raised you?”; (6) “Were you ever mugged, held up, or threatened with a weapon?”; (7) “Did someone very close to you ever die unexpectedly; for example, they were killed in an accident, murdered, committed suicide, or had a fatal heart attack at a young age?”; (8) “Did you ever have a son or daughter who had a life-threatening illness or injury?”; (9) “Did anyone very close to you ever have an extremely traumatic experience, like being kidnapped, tortured, or raped?”; (10) “Did you ever do something that accidentally led to the serious injury or death of another person?”; (11) “Did you ever on purpose either seriously injure, torture, or kill another person?”; (12) “Did you ever experience any other extremely traumatic or life-threatening event that I haven’t asked about yet?”; and (13) “Did you ever have a traumatic event that you didn’t report because you didn’t want to talk about it?” Five items were regarded as refugee-related: (1) “Were you ever an unarmed civilian in a place where there was a war, revolution, military coup, or invasion?” (2) “Were you ever involved in a major natural disaster, like a devastating flood, hurricane, or earthquake?” (3) “Were you ever in a man-made disaster, like a fire started by a cigarette or a bomb explosion?” (4) “Did you ever see someone being badly injured or killed, or unexpectedly see a dead body?”; and (5) “Did you ever see atrocities or carnage such as mutilated bodies or mass killings?” (eAppendix 1 in the Supplement).^6^ Items were coded 1 for yes, and 0 for no for lifetime exposure, generating 2 counts for general and refugee-related TEs. Because of skewed distributions in each count, we grouped scores into hierarchical categories. General TEs were grouped into 4 categories (0, 1, 2-3, and ≥4); refugee-related TEs were grouped into 3 categories (0, 1, and ≥2).

#### Refugee-Specific Question

Women were asked, “Were you ever a refugee—that is, did you ever flee from your home to a foreign country or place to escape danger or persecution?” Answers were coded as 1 for yes and 0 for no.

#### Intimate Partner Violence

The World Health Organization measure includes items inquiring into physical, psychological, and sexual violence perpetrated by an intimate partner over the past 12 months.^15,16^ Cultural experts advised against including explicit sexual abuse items. We assigned women to 3 hierarchical and mutually exclusive categories: (1) no IPV or low respect or regard only; (2) severe psychological IPV alone; and (3) physical IPV.^8^ This means that any woman with physical IPV was assigned to the highest category, whether or not she reported psychological abuse or endorsed low respect or regard. Women who were placed in the second category reported psychological abuse and could report low respect or regard but not physical IPV. The lowest category contains the women who did not report physical or psychological IPV but may have reported low respect or regard.

#### Social Support

Social support was assessed according to the number of reliable persons (family or friends) in whom the woman could confide if she confronted serious problems. Based on the distribution, 3 hierarchical categories were generated according to the number of available confidantes: 5 or more, 3 to 4, and 2 or fewer (eAppendix 2 in the Supplement).

#### Finance-Related Stressors

A composite index of finance-related stressors comprised items relating to difficulties such as paying bills and affording enough food and heating (score range, 0-7). Because of skewed scores, we generated 3 hierarchical categories (0, 1-2, and ≥3).

### Statistical Analysis

Descriptive statistics and χ^2^ tests are reported for bivariate analyses examining putative risk factors of MDD for the 3 samples. Variables included sociodemographic characteristics, self-identification as a refugee (given to all women born in conflict-affected countries), general TEs, refugee-relevant TEs, IPV, social support, and finance-related stressors. Statistically significant indices were tested in preliminary multiple regression analyses to obtain the best model for MDD.^17^ For the preliminary screening regression models, statistical significance was set at *P* < .05 for 2-tailed tests. Adjusted odds ratios (aORs) with 95% CIs are presented for the final model. The analysis was performed using SPSS version 25 (IBM).^18^

## Results

### Participants

Of all 1574 eligible women, 1335 (84.8%) were interviewed, including 650 women born in Australia (48.7%) and 685 from conflict-affected countries (51.3%). Of the 685 participants from conflict-affected countries, 289 women self-identified as refugees (42.2%). Women primarily cited time constraints for declining to participate. The mean (SD) age for women born in Australia was 29.0 (5.5) years; for women from conflict-affected countries, it was 29.7 (5.4) years (Table 1). Most women (1215 [91.0%]) across groups were married or lived with an intimate partner. The most highly represented countries of origin in the women from conflict-affected regions were Iraq (260 [38.0%]), Lebanon (125 [18.2%]), Sri Lanka (71 [10.4%]) and Sudan (66 [9.6%]). Only 1 in 5 women who identified as refugees (60 [20.8%]) held a university degree; for women from other conflict-affected backgrounds and women born in the host nation, 153 (38.6%) and 193 (29.7%), respectively, held a university degree. Most women from all groups (1050 [78.7%]) lived with a partner and children with or without other persons. Most women who identified as refugees (190 [65.7%]) lived in rental accommodation, whereas a minority of women from the host nation (241 [37.1%]) and from other conflict-affected backgrounds (177 [44.7%]) lived under these circumstances. Only one-quarter of women who identified as refugees (71 [24.6%]) were employed compared with 127 women from other conflict-affected backgrounds (32.1%) and 383 for women born in the host nation (58.1%) (Table 1).

**Table 1.** Sociodemographic Characteristics

Characteristic	No. (%)			
	Women Born in the Host Nation (n = 650)	All Women From Conflict-Affected Countries^a^ (n = 685)	Women Who Identified as Refugees^b^ (n = 289)	Women from Other Conflict-Affected Backgrounds (n = 396)
Age group, y				
≤24	153 (23.5)	122 (17.8)	59 (20.4)	63 (15.9)
25-34	381 (58.6)	423 (61.8)	157 (54.3)	266 (67.2)
≥35	116 (17.8)	140 (20.4)	73 (25.3)	67 (16.9)
Age, mean (SD), y	29.0 (5.5)	29.7 (5.4)	30.0 (5.8)	29.6 (5.1)
Marital status				
Married/domestic partnership	566 (87.1)	649 (94.7)	268 (92.7)	381 (96.2)
Separated/divorced/other	84 (12.9)	36 (5.3)	21 (7.3)	15 (3.8)
Highest level of educational attainment				
No postschool qualification	286 (44.0)	350 (51.1)	178 (61.6)	172 (43.4)
Diploma and vocational education	171 (26.3)	122 (17.8)	51 (17.6)	71 (17.9)
University degree	193 (29.7)	213 (31.1)	60 (20.8)	153 (38.6)
Family composition of household				
1-Parent family with dependent children/others	65 (10.0)	49 (7.1)	20 (6.9)	29 (7.3)
2-Person family with or without dependent children/others	494 (76.0)	556 (81.2)	227 (78.5)	329 (83.1)
Multiple-person family with or without dependent children	91 (14.0)	80 (11.7)	42 (14.5)	38 (9.6)
Housing status				
Owner without a mortgage	39 (6.0)	30 (4.4)	9 (3.1)	21 (5.3)
Owner with a mortgage	252 (38.8)	212 (30.9)	74 (25.6)	138 (34.8)
Renter	241 (37.1)	367 (53.6)	190 (65.7)	177 (44.7)
Boarder and others	118 (18.2)	76 (11.1)	16 (5.5)	60 (15.2)
Employment status				
Employed	383 (58.1)	198 (28.9)	71 (24.6)	127 (32.1)
Unemployed and others	267 (41.1)	487 (71.1)	218 (75.4)	269 (67.9)

^a^ Country of birth for women with conflict-affected backgrounds: Iraq, 260 (38.0%); Lebanon, 125 (18.2%); Sudan, 66 (9.6%); Syria, 30 (4.4%); Egypt, 29 (4.2%); Afghanistan, 13 (1.9%); Sri Lanka, 71 (10.4%); and India, Pakistan, and others, 91 (13.3%).

^b^ Women who identified as refugees grouped according to a self-identifying item. Women who answered no were classified as women from other conflict-affected backgrounds.

### Indices of Adversity

#### Traumatic Events

Women who identified as refugees reported higher exposure to 2 to 3 (67 [23.2%]) and 4 or more (19 [6.6%]) general TEs compared with women born in Australia (103 [15.8%] and 21 [3.2%], respectively) and women from other conflict-affected backgrounds (45 [11.4%] and 6 [1.5%], respectively) (Table 2). Women who identified as refugees also reported higher exposure to 1 (147 [50.9%]) and 2 or more (97 [33.6%]) refugee-related TEs compared with women born in Australia (86 [13.2%] and 20 [3.1%], respectively) and women from other conflict-affected backgrounds (114 [28.8%] and 47 [11.9%], respectively). Differences between all groups in relation to both categories of trauma were statistically significant (Table 2; eTable 1 in the Supplement).

**Table 2.** Traumatic Events, Refugee-Specific Characteristics, IPV, Social Support, and Finance-Related Stressors

Characteristic	No. (%)			
	Women Born in the Host Nation (n = 650)	All Women From Conflict-Affected Countries^a^ (n = 685)	Women Who Identified as Refugees^b^ (n = 289)	Women From Other Conflict-Affected Backgrounds (n = 396)
General TE counts^c^				
0	344 (52.9)	336 (49.1)	108 (37.4)	228 (57.6)
1	182 (28.0)	212 (30.9)	95 (32.9)	117 (29.5)
2-3	103 (15.8)	112 (16.4)	67 (23.2)	45 (11.4)
≥4	21 (3.2)	25 (3.6)	19 (6.6)	6 (1.5)
Refugee-related TE counts^d^				
0	544 (83.7)	280 (40.9)	45 (15.6)	235 (59.3)
1	86 (13.2)	261 (38.1)	147 (50.9)	114 (28.8)
≥2	20 (3.1)	144 (21.0)	97 (33.6)	47 (11.9)
Refugee-specific characteristics				
Duration in Australia				
Arrived before 2001	NA	103 (15.0)	34 (11.8)	69 (17.4)
Arrived 2001-2010	NA	267 (39.0)	130 (45.0)	137 (34.6)
Arrived 2011-2014	NA	229 (33.4)	88 (30.4)	141 (35.6)
Arrived in 2015 or later	NA	86 (12.6)	37 (12.8)	49 (12.4)
Professional role change				
Yes	NA	415 (60.6)	181 (62.6)	234 (59.1)
No/other	NA	270 (39.4)	108 (37.4)	162 (40.9)
Adapted to Australian way of doing things				
Not at all to moderate	NA	278 (40.6)	117 (40.5)	161 (40.7)
Somewhat/very much	NA	407 (59.4)	172 (59.5)	235 (59.3)
IPV				
No IPV and/or low respect	482 (74.2)	381 (55.6)	141 (48.8)	240 (60.6)
Severe psychological IPV	133 (20.5)	259 (37.8)	124 (42.9)	135 (34.1)
Physical IPV	35 (5.4)	45 (6.6)	24 (8.3)	21 (5.3)
No. of friends/family members who can be relied on for serious problems^e^				
≥5	297 (45.7)	120 (17.5)	36 (12.5)	84 (21.2)
3-4	245 (37.7)	273 (39.9)	125 (43.3)	148 (37.4)
≤2	108 (16.6)	292 (42.6)	128 (44.3)	164 (41.4)
No. of finance-related stressors				
0	498 (76.6)	427 (62.3)	154 (53.3)	273 (68.9)
1-2	111 (17.1)	146 (21.3)	70 (24.2)	76 (19.2)
≥3	41 (6.3)	112 (16.4)	65 (22.5)	47 (11.9)

Abbreviations: IPV, intimate partner violence; NA, not applicable; TE, traumatic event.

^a^ Country of birth for women with conflict-affected backgrounds: Iraq, 260 (38.0%); Lebanon, 125 (18.2%); Sudan, 66 (9.6%); Syria, 30 (4.4%); Egypt, 29 (4.2%); Afghanistan, 13 (1.9%); Sri Lanka, 71 (10.4%); and India, Pakistan, and others, 91 (13.3%).

^b^ Women who identified as refugees grouped according to a self-identifying item. Women who answered no were classified as women from other conflict-affected backgrounds.

^c^ General TE counts included 13 items, as specified in the Methods. Each item was coded 1 for yes and 0 for no.

^d^ Refugee-related TEs included 5 items, as specified in the Methods. Each item was coded 1 for yes and 0 for no.

^e^ Number of friends and family members who can be relied on for serious problems based on 2 items, as specified in the Methods. Each item was coded 0 for 5 or more members, 1 for 3 to 4 members, and 2 for 2 or fewer members.

#### Intimate Partner Violence (IPV)

Women who identified as refugees reported higher rates of psychological IPV compared with women born in the host nation (124 [42.9%] vs 133 [20.5%]; *P* < .001) and compared with women from other conflict-affected backgrounds (124 [42.9%] vs 135 [34.1%]; *P* = .002) (Table 2; eTable 1 in the Supplement). There were no statistical differences in the prevalence of physical IPV across the groups.

#### Social Support

Women who identified as refugees were less likely to identify 5 or more supportive family members or friends compared with women born in the host nation (36 [12.5%] vs 297 [45.7%]; *P* < .001) and women from other conflict-affected backgrounds (36 [12.5%] vs 84 [21.2%]; *P* = .003) (Table 2; eTable 1 in the Supplement). Women who identified as refugees were more likely to report having 2 or fewer supportive family members (128 [44.3%]) compared with women born in the host nation (108 [16.6%]; *P* < .001). Of women from other conflict-affected backgrounds, 164 (41.4%) reported having 2 or fewer supportive family members, but comparison with women who identified as refugees was not significant (Table 2; eTable 1 in the Supplement).

#### Finance-Related Stressors

A greater proportion of women who identified as refugees (65 [22.5%]) reported experiencing 3 or more finance-related stressors. Of women born in the host nation, 41 (6.3%; *P* < .001) reported experiencing 3 or more finance-related stressors, and 47 women from other conflict-affected backgrounds (11.9%; *P* < .001) reported experiencing 3 or more financial stressors.

#### Prevalence of MDD

Women who identified as refugees had the highest prevalence of MDD (94 [32.5%]) followed by women from other conflict-affected backgrounds (78 [19.7%]) and women born in the host nation (94 [14.5%]) (*P* < .001). Notably, women who identified as refugees had twice the prevalence of MDD of women born in the host nation (Table 3).

**Table 3.** Prevalence of MDD and Sociodemographic Characteristics

Sociodemographic Characteristic	Prevalence of MDD, No. (%)^a^			
	Women Born in the Host Nation (n = 650)	All Women From Conflict-Affected Countries^b^ (n = 685)	Women Who Identified as Refugees^c^ (n = 289)	Women From Other Conflict-Affected Backgrounds (n = 396)
All women	94/650 (14.5)	172/685 (25.1)	94/289 (32.5)	78/396 (19.7)
Age group, y				
<25	29/153 (19.0)	34/122 (27.9)	22/59 (37.3)	12/63 (19.0)
25-34	49/381 (12.9)	103/423 (24.3)	52/157 (33.1)	51/266 (19.2)
≥35	16/116 (13.8)	35/140 (25.0)	20/73 (27.4)	15/67 (22.4)
*P* value^d^	.19	.73	.47	.83
Marital status				
Married/domestic partnership	71/566 (12.5)	158/649 (24.3)	87/268 (32.5)	71/381 (18.6)
Separated/divorced/other	23/84 (27.4)	14/36 (38.9)	7/21 (33.3)	7/15 (46.7)
*P* value^d^	.001	.04	.94	.02
Highest level of educational attainment				
No postschool qualification	45/286 (15.7)	99/350 (28.3)	60/178 (33.7)	39/172 (22.7)
Diploma and vocational education	29/171 (17.0)	28/122 (23.0)	12/51 (23.5)	16/71 (22.5)
University degree	20/193 (10.4)	45/213 (21.1)	22/60 (36.7)	23/153 (15.0)
*P* value^d^	.15	.14	.29	.18
Family composition of household				
1-Parent family with dependent children/others	18/65 (27.7)	17/49 (34.7)	6/20 (30.0)	11/29 (37.9)
2-Person family with or without dependent children/others	60/494 (12.1)	136/556 (24.5)	76/227 (33.5)	60/329 (18.2)
Multiple person family with or without dependent children	16/91 (17.6)	19/80 (23.8)	12/42 (28.6)	7/38 (18.4)
*P* value^d^	.002	.35	.80	.06
Housing status				
Owner without a mortgage	8/39 (20.5)	5/30 (16.7)	2/9 (22.2)	3/21 (14.3)
Owner with a mortgage	31/252 (12.3)	47/212 (22.2)	18/74 (24.3)	29/138 (21.0)
Renter	41/241 (17.0)	107/367 (29.2)	73/190 (38.4)	34/177 (19.2)
Boarder and others	14/118 (11.9)	13/76 (17.1)	1/16 (6.3)	12/60 (20.0)
*P* value^d^	.26	.048	.01	.90
Employment status				
Employed	39/383 (10.2)	30/198 (15.2)	14/71 (19.7)	16/127 (12.6)
Unemployed and others	55/267 (20.6)	142/487 (29.2)	80/218 (36.7)	62/269 (23.0)
*P* values^d^	<.001	<.001	.01	<.001

Abbreviation: MDD, major depressive disorder.

^a^ Mini-International Neuropsychiatric Interview for the *Diagnostic and Statistical Manual of Mental Disorders* (Fourth Edition) was used to diagnose MDD, as described in the Methods. Each item was coded 1 for yes and 0 for no. Women who answered yes for either item 1 or 2 and answered yes for at least 3 other items were classified as having MMD.

^b^ Country of birth for women with conflict-affected backgrounds: Iraq, 260 (38.0%); Lebanon, 125 (18.2%); Sudan, 66 (9.6%); Syria, 30 (4.4%); Egypt, 29 (4.2%); Afghanistan, 13 (1.9%); Sri Lanka, 71 (10.4%); and India, Pakistan, and others, 91 (13.3%).

^c^ Women who identified as refugees grouped according to a self-identifying item. Women who answered no were classified as women from other conflict-affected backgrounds.

^d^ *P* values derived from χ^2^ test conducted to examine the significant variation of prevalence of MDD for each factor within the women born in host nation, all women from conflict-affected countries, women who self-identified as refugees, and women from other conflict-affected backgrounds. *P* values represent the significance level within the group.

### Bivariate Correlates of MDD

Table 3 reports the relevant findings. The absence of a domestic partner was associated with MDD for women born in the host nation and women from other conflict-affected backgrounds but not refugee women. Major depressive disorder was only associated with living in a 1-parent household among women born in the host nation. Not owning a home (eg, renting) was associated with MDD among refugee women only. Unemployment was associated with MDD across all categories of women (Table 3).

### Adversity Indices

The general TE count was directly associated with MDD for all categories of women (Table 4). Among women who were exposed to 4 or more TEs, 12 of 19 self-identified refugees (63%) and 7 of 21 born in the host country (33%) experienced MDD (*P* = .20). The number of women from other conflict-affected backgrounds exposed to this highest level of trauma was too low to make meaningful comparisons with other groups of women (n = 2). In relation to exposure to 2 or more refugee-relevant TEs, women who identified as refugees had a higher prevalence of MDD compared with women from other conflict-affected backgrounds (45 [46.4%] vs 13 [27.7%]; *P* = .03). Comparisons with women born in the host nation (n = 6) were not meaningful, given the low level of exposure to refugee-related TEs in this group

**Table 4.** Prevalence of MDD by TEs, Refugee-Specific Characteristics, IPV, Social Support, and Finance-Related Stressors

Characteristic	Prevalence of MDD, No./Total No. (%)^a^			
	Women Born in the Host Nation (n = 650)	All Women From Conflict-Affected Countries^b^ (n = 685)	Women Who Identified as Refugees^c^ (n = 289)	Women From Other Conflict-Affected Backgrounds (n = 396)
TEs				
General TE counts^d^				
0	39/344 (11.3)	58/336 (17.3)	26/108 (24.1)	32/228 (14.0)
1	23/182 (12.6)	57/212 (26.9)	31/95 (32.6)	26/117 (22.2)
2-3	25/103 (24.3)	43/112 (38.4)	43/67 (37.3)	18/45 (40.0)
≥4	7/21 (33.3)	14/25 (56.0)	12/19 (63.2)	2/6 (33.3)
*P* value^e^	.003	.001	.006	.001
Refugee-related TE counts^f^				
0	72/544 (13.2)	46/280 (16.4)	10/45 (22.2)	36/235 (15.3)
1	16/86 (18.6)	68/261 (26.1)	39/147 (26.5)	29/114 (25.4)
≥2	6/20 (30.0)	58/144 (40.3)	45/97 (46.4)	13/47 (27.7)
*P* value^e^	.09	.001	.001	.03
Refugee-specific characteristics				
Duration in Australia				
Arrived before 2001	NA	22/103 (21.4)	12/34 (35.3)	10/69 (14.5)
Arrived 2001-2010	NA	65/267 (24.3)	34/130 (26.2)	31/137 (22.6)
Arrived 2011-2014	NA	56/229 (24.5)	30/88 (34.1)	26/141 (18.4)
Arrived in 2015 or later	NA	29/86 (33.7)	18/37 (48.6)	11/49 (22.4)
*P* value^c^	NA	.20	.07	.51
Professional role change				
Yes	NA	110/415 (26.5)	56/181 (30.9)	54/234 (23.1)
No/others	NA	62/270 (23.0)	38/108 (35.2)	24/162 (14.8)
*P* value^e^	NA	.30	.52	.05
Adapted to Australian way of doing things				
Not at all to moderate	NA	88/278 (31.7)	44/117 (37.6)	44/161 (27.3)
Somewhat/very much	NA	84/407 (20.6)	50/172 (29.1)	34/235 (14.5)
*P* value^e^	NA	.001	.13	.002
IPV				
No IPV and/or low respect	52/482 (10.8)	63/381 (16.5)	38/141 (27.0)	25/240 (10.4)
Severe psychological IPV	26/133 (19.5)	81/259 (31.3)	42/124 (33.9)	39/135 (28.9)
Physical IPV	16/35 (45.7)	28/45 (62.2)	14/24 (58.3)	14/21 (66.7)
*P* value^e^	.001	.001	.009	.001
No. of friends/family members who can be relied on for serious problems^f^				
≥5	33/297 (11.1)	13/120 (10.8)	8/36 (22.2)	5/84 (6.0)
3-4	38/245 (15.5)	67/273 (24.5)	40/125 (32.0)	27/148 (18.2)
≤2	23/108 (21.3)	92/292 (31.5)	46/128 (35.9)	46/164 (28.0)
*P* value^c^	.03	.001	.30	.001
No. of finance-related stressors				
0	55/498 (11.0)	68/427 (15.9)	33/154 (21.4)	35/273 (12.8)
1-2	25/111 (22.5)	56/146 (38.4)	27/70 (38.6)	29/76 (38.2)
≥3	14/41 (34.1)	48/112 (42.9)	34/65 (52.3)	14/47 (29.8)
*P* value^c^	.001	.001	.001	.001

Abbreviations: IPV, intimate partner violence; MDD, major depressive disorder; NA, not applicable; TE, traumatic event.

^a^ Mini-International Neuropsychiatric Interview for the *Diagnostic and Statistical Manual of Mental Disorders* (Fourth Edition) was used to diagnose MDD, as specified in the Methods. Each item was coded 1 for yes and 0 for no. Women who answered yes for either item 1 or 2 and answered yes for at least 3 other items were classified as having MDD.

^b^ Country of birth for women with conflict-affected backgrounds: Iraq, 260 (38.0%); Lebanon, 125 (18.2%); Sudan, 66 (9.6%); Syria, 30 (4.4%); Egypt, 29 (4.2%); Afghanistan, 13 (1.9%); Sri Lanka, 71 (10.4%); and India, Pakistan, and others, 91 (13.3%).

^c^ Women who identified as refugees grouped according to a self-identifying item. Women who answered no were classified as women from other conflict-affected backgrounds.

^d^ General TE counts included 13 items, as specified in the Methods. Each item was coded 1 for yes and 0 for no.

^e^ *P* values derived from χ^2^ test conducted to examine the significant variation of prevalence of MDD for each factor within the women born in host nation, all women from conflict-affected countries, women who self-identified as refugees, and women from other conflict-affected backgrounds. *P* values represent the significance level within the group.

^f^ Refugee-related TEs included 5 items, as specified in the Methods. Each item was coded 1 for yes and 0 for no.

^g^ No. of friends/family members who can be relied on for serious problems based on 2 items, as specified in the Methods. Each item was coded 0 for 5 or more members, 1 for 3 to 4 members, and 2 for 2 or fewer members.

Intimate partner violence was associated with MDD across all 3 groups of women. A total of 42 women who identified as refugees (34%), 39 women from other conflict-affected backgrounds (29%), and 26 women born in the host nation (20%) who reported psychological IPV had MDD. Among women who identified as refugees experiencing physical IPV, 14 had MDD (58%); among women born in the host nation, 16 had MDD (46%); and among women from other conflict-affected backgrounds, 14 had MDD (67%) (Table 4).

There was an association of lower levels of social support with MDD prevalence for all categories of women (Table 4). At the most extreme level of 3 or more finance-related stressors, 34 women who identified as refugees (52%) compared with 14 women born in the host nation (34%; *P* = .07) and 14 women from other conflict-affected backgrounds (30%; *P* = .02) experienced MDD.

In summary, the findings indicate that all indices of TEs and adversity assessed were associated with MDD in most subgroups of women. The refugee-relevant TEs were strongly associated with identifying as a refugee, as anticipated. In addition, women who identified as refugees reported the highest level of exposure to general TEs and all forms of psychosocial adversity, although, in some instances, exposure did not differ from women from other conflict-affected backgrounds.

### Multiple Logistic Regression Analysis

Multiple logistic regression analysis was conducted to obtain a comprehensive model of MDD. A specific aim was to assess whether self-identification as a refugee contributed to MDD prevalence even when the overall variance of other risk factors was included in the model. Preliminary testing of the model excluded most sociodemographic variables, such as age and marital status. Importantly, a 2-way index categorizing women as born in the host nation vs women from all conflict-affected backgrounds did not contribute to the model. Only when a 3-way categorization was included (women who identified as refugees, women from other conflict-affected backgrounds, and women born in the host nation) was the refugee category associated with MDD. In addition, preliminary testing indicated, as anticipated, a high degree of collinearity of the refugee self-identification item with refugee-related TEs (*r* = 0.50; 95% CI, 0.46-0.50; *P* < .001). Given the centrality of the refugee item to the aims of the study, we retained that item in the final model and excluded the index of refugee-related TEs. As can be seen from Table 5 and eTable 2 in the Supplement, the following risk factors were significant in the final logistic regression: identifying as a refugee (aOR, 1.57; 95% CI, 1.07-2.30); unemployment (aOR, 1.44; 95% CI, 1.03-2.03); exposure to 2 or more general TEs (aOR, 2.13; 95% CI, 1.47-3.08); severe psychological IPV (aOR, 1.62; 95% CI, 1.18-2.23); physical IPV (aOR, 4.64; 95% CI, 2.78-7.74); low levels of support (≤2 persons available: aOR, 1.78; 95% CI, 1.16-2.72); and finance-related stressors (1-2 stressors: aOR, 1.87; 95% CI, 1.31-2.66; ≥3 stressors: aOR, 2.17; 95% CI, 1.43-3.29).

**Table 5.** Significant Factors Associated With MDD From Multiple Logistic Regression Analysis for Combined Sample of 1335 Women

Significant Factor^a^	Sample Size, No. (%)	Prevalence of MDD, No. (%)^b^	Adjusted OR (95% CI)^c^	*P* Value for Adjusted OR	*P* Value^d^
All women	1335 (100)	266 (19.9)	NA	NA	NA
Strictly defined refugee status					
Women born in host nation	650 (48.7)	94 (14.5)	1 [Reference]^f^	NA	<.001
Women who identified as refugees^e^	289 (21.6)	94 (32.5)	1.57 (1.07-2.30)	.02	
Women from other conflict-affected backgrounds	396 (29.7)	78 (19.7)	1.10 (0.76-1.60)	.61	
Employment status					
Employed	581 (43.5)	69 (11.9)	1 [Reference]^f^	NA	<.001
Unemployed	754 (56.5)	197 (26.1)	1.44 (1.03-2.03)	.04	
General TEs^g^					
0	680 (50.9)	97 (14.3)	1 [Reference]^f^	NA	<.001
1	394 (29.5)	80 (20.3)	1.28 (0.91-1.81)	.16	
≥2	261 (19.6)	89 (34.1)	2.13 (1.47-3.08)	<.001	
IPV					
No IPV and/or low respect	863 (64.6)	115 (13.3)	1 [Reference]^f^	NA	<.001
Severe psychological IPV	392 (29.4)	107 (27.3)	1.62 (1.18-2.23)	.003	
Physical IPV	80 (6.0)	44 (55.0)	4.64 (2.78-7.74)	<.001	
No. of friends /family members who can be relied on for serious problems^h^					
≥5	417 (31.2)	46 (11.0)	1 [Reference]^f^	NA	<.001
3-4	518 (38.8)	105 (20.3)	1.35 (0.90-2.04)	.15	
≤2	400 (30.0)	115 (28.8)	1.78 (1.16-2.72)	.008	
No. of finance related stressors					
0	925 (69.3)	123 (13.3)	1 [Reference]^f^	NA	<.001
1-2	257 (19.3)	81 (31.5)	1.87 (1.31-2.66)	.001	
≥3	153 (11.5)	62 (40.5)	2.17 (1.43-3.29)	<.001	

Abbreviations: IPV, intimate partner violence; MDD, major depressive disorder; NA, not applicable; OR, odds ratio; TEs, traumatic events.

^a^ Factors included in multiple logistic regression model were found to be statistically significant (*P* < .05) in stepwise multiple logistic regression analysis for women born in host nation and all women from conflict-affected countries.

^b^ Mini-International Neuropsychiatric Interview for the *Diagnostic and Statistical Manual of Mental Disorders* (FourthEdition) was used to diagnose MDD. Women who answered yes for either item 1 or 2 and answered yes for at least 3 other items were classified as having MMD.

^c^ The outcome variable MDD for multiple logistic regression model was coded as 1 for depressed and 0 for not depressed.

^d^ *P* values derived from χ^2^ test conducted to examine the significant variation of prevalence of MDD for each factor within the women born in host nation, all women from conflict-affected countries, women who self-identified as refugees, and women from other conflict-affected backgrounds. *P* values represent the significance level within the group.

^e^ Women who identified as refugees grouped according to a self-identifying item, as specified in the Methods. Women who answered no were classified as women from other conflict-affected backgrounds.

^f^ Used as reference category in logistic regression analysis.

^g^ General TE counts included 13 items, as specified in the Methods. Each item was coded 1 for yes and 0 for no.

^h^ Number of friends and family members who can be relied on for serious problems based on 2 items, as specified in the Methods.

## Discussion

In total, 94 women who identified as refugees (32.5%), 78 women from other conflict-affected backgrounds (19.7%), and 94 women born in the host nation (14.5%) received a diagnosis of MDD. Although trauma exposure and other forms of psychosocial adversity were universally relevant in association with MDD, women who identified as refugees reported higher exposure to these experiences. Moreover, even when these factors were included in a multivariate analysis, the single item of women self-identifying as refugees continued to play a role in predicting MDD risk. It was highly relevant that the single question related to refugee self-identification reflected a high risk that the woman had experienced the 5 refugee-relevant TEs.

### Strengths and Limitations

Strengths of the study are that it is, to our knowledge, the first systematic inquiry of its kind among women from conflict-affected countries attending antenatal clinics in a high-income resettlement country. The sample size was substantial and the response rate high. Field workers were women from the same cultural and linguistic backgrounds as participants. We recruited host-nation and conflict-affected samples from the same locations over the same period. Because of the uneven size of each population, we used a computerized randomization method to select the subsample of women born in the host nation, an approach that should not have introduced systematic bias. We selected widely used instruments to assess MDD and IPV and followed consensus guidelines in adapting the measures to each language and cultural group. We applied a structured diagnostic measure rather than screening instruments, which tend to overestimate the prevalence of MDD.^3^ We selected MDD as the focus of the study because it is the disorder most clearly associated with high levels of functional impairment across populations worldwide.

Our study had limitations. Our deliberate strategy to focus on public health clinics where women from conflict-affected countries concentrate may mean that the findings are not fully generalizable to women attending private clinics or those living in low-density migrant areas. Retrospective distortions, gaps in memory, and reluctance to divulge sensitive information (eg, related to IPV) are acknowledged possibilities that may lead to inaccuracies in reporting of past events. It is difficult to determine whether these influences led to overreporting or underreporting of adversities. Additionally, the cross-sectional nature of the analyses cautions against drawing definitive causal explanations from the findings.

## Conclusions

Our findings have important implications for identifying pregnant women with conflict-affected and refugee backgrounds at risk of psychosocial and mental health problems. In general, the findings confirm a higher prevalence of MDD among women who identify as refugees compared with women from other conflict-affected backgrounds and women born in the host nation. All women from conflict-affected backgrounds had a higher prevalence of MDD compared with those born in the host nation. We confirmed our hypothesis that there are a set of risk factors of MDD that affect pregnant women from diverse backgrounds and countries of origin, further justifying screening for these indices. A novel finding is that a single self-identifying item defines a subpopulation of women at high risk of exposure to traumas (related to past conflict and IPV), psychosocial difficulties, and MDD. In that sense, without a detailed inquiry into potentially sensitive information, front-line workers in antenatal clinics can use the refugee item as a signpost that may warrant referring women for further screening, for example by a social worker who can inquire into the trauma background, ongoing psychosocial status, and possible presence of MDD.

Our findings also have more general implications for understanding the global literature relating to the adverse mental health effects of mass conflict. Nationally representative mental health surveys conducted in countries exposed to repeated warfare and mass conflict^6^ have yielded prevalence rates of common mental disorders, such as MDD, that show few differences from rates from non–conflict-affected countries. The error would be to interpret these findings as suggesting that mass conflict does not increase the risk of mental disorders, such as depression. The appropriate inference should be that there is substantial variability in exposure to trauma and psychosocial adversity within conflict-affected countries, extending to populations who migrate from these settings. Importantly, self-identification of refugee status may be a relatively accurate indicator of the subpopulation exposed to high levels of trauma and psychosocial adversity as well as increased risk of mental disorders, such as MDD. From a pragmatic perspective, our findings suggest that it is not necessary to offer direct psychosocial or mental health interventions for all women migrating from conflict-affected countries, which would not be feasible in any event, given the shortage of resources and skills available to match the assumed needs. Instead, it may be that simple screening questions, such as whether a woman perceives herself to be a refugee, may assist in distinguishing subpopulations at the greatest risk of psychosocial adversity and mental disorders such as MDD.

## Notes

Supplement. **eAppendix 1.** General and Refugee-Relevant Traumatic Events **eAppendix 2.** Family and Social Support Items **eTable 1.** Prevalence of Predictors Used in Logistic Regression Analysis by Women Born in Host Nation, Women Who Identified as Refugees, and Women From Other Conflict-Affected Backgrounds Results of Quality Assessment per Study **eTable 2.** Adjusted Odds Ratios (OR) With 95% CIs From Multiple Logistic Regression Analysis for Combined Sample of Women Born in Host Nation and All Women from Conflict-Affected Countries (n = 1335) Controlling for TE Counts
