# Supplementary material for: Prevalence and Risk Factors of Major Depressive Disorder Among Women at Public Antenatal Clinics From Refugee, Conflict-Affected, and Australian-Born Backgrounds
Source: JAMA Netw Open. 2019 May 3;2(5):e193442. doi: 10.1001/jamanetworkopen.2019.3442 (PMC6503483; doi:10.1001/jamanetworkopen.2019.3442)
Supplement: Supplement. — eAppendix 1. General and Refugee-Relevant Traumatic Events eAppendix 2. Family and Social Support Items eTable 1. Prevalence of Predictors Used in Logistic Regression Analysis by Women Born in Host Nation, Women Who Identified as Refugees, and Women From Other Conflict-Affected Backgrounds Results of Quality Assessment per Study eTable 2. Adjusted Odds Ratios (OR) With 95% CIs From Multiple Logistic Regression Analysis for Combined Sample of Women Born in Host Nation and All Women from Conflict-Affected Countries (n = 1335) Controlling for TE Counts [file jamanetwopen-2-e193442-s001.pdf]

Supplementary Online Content

Rees SJ, Fisher JR, Steel Z, et al. Prevalence and risk factors of major depressive disorder among women at public antenatal clinics from refugee, conflict-affected, and Australian-born backgrounds. *JAMA Netw Open*. 2019;2(5):e193442. doi:10.1001/jamanetworkopen.2019.3442

eAppendix 1. General and Refugee-Relevant Traumatic Events
eAppendix 2. Family and Social Support Items
eTable 1. Prevalence of Predictors Used in Logistic Regression Analysis by Women Born in Host Nation, Women Who Identified as Refugees, and Women From Other Conflict-Affected Backgrounds Results of Quality Assessment per Study
eTable 2. Adjusted Odds Ratios (OR) With 95% CIs From Multiple Logistic Regression Analysis for Combined Sample of Women Born in Host Nation and All Women from Conflict-Affected Countries (n = 1335) Controlling for TE counts

This supplementary material has been provided by the authors to give readers additional information about their work.

© 2019 Rees SJ et al. *JAMA Network Open.*

eAppendix 1. General and Refugee-Relevant Traumatic Events

A total of 19 Potential Traumatic Events (PTEs) items were produced from the three following item categories: General Traumatic Events (General TEs; 13 items), Refugee-relevant Traumatic Events (Refugee-relevant TEs; 5 items), and a Refugee Self-identification item (1 item).

General TEs

(1) ‘Were you ever kidnapped or held captive?’; (2) ‘Were you ever involved in a life-threatening automobile accident?’; (3) ‘Did you ever have any other life-threatening accident, including on your job?’; (4) ‘Did you ever have a life-threatening illness?’; (5) ‘As a child, were you ever badly beaten up by your parents or the people who raised you?’; (6) ‘Were you ever mugged, held up, or threatened with a weapon?’; (7) ‘Did someone very close to you ever die unexpectedly; for example, they were killed in an accident, murdered, committed suicide, or had a fatal heart attack at a young age?’; (8) ‘Did you ever have a son or daughter who had a life-threatening illness or injury?’; (9) ‘Did anyone very close to you ever have an extremely traumatic experience, like being kidnapped, tortured or raped?’; (10) ‘Did you ever do something that accidentally led to the serious injury or death of another person?’; (11) ‘Did you ever on purpose either seriously injure, torture, or kill another person?’; (12) ‘Did you ever experience any other extremely traumatic or life-threatening event that I haven’t asked about yet?’; (13) ‘Did you ever have a traumatic event that you didn’t report because you didn’t want to talk about it?’ (each item coded yes=1, no=0).

Refugee-relevant TEs

(1) ‘Were you ever an unarmed civilian in a place where there was a war, revolution, military coup or invasion?’; (2) ‘Were you ever involved in a major natural disaster, like a devastating

© 2019 Rees SJ et al. *JAMA Network Open.*

flood, hurricane, or earthquake?' (3) 'Were you ever in a man-made disaster, like a fire started by a cigarette, or a bomb explosion?'; (4) 'Did you ever see someone being badly injured or killed, or unexpectedly see a dead body?'; (5) 'Did you ever see atrocities or carnage such as mutilated bodies or mass killings?' (each item coded yes=1, no=0).

Refugee Self-identification item

(1) 'Were you ever a refugee – that is, did you ever flee from your home to a foreign country or place to escape danger or persecution?' Among the women from Conflict-countries, those who answered 'yes' are classified as 'Self-identifying Refugee Women' (Refugee women) and remaining are classified as 'Other Conflict-affected Background Women'.

© 2019 Rees SJ et al. *JAMA Network Open.*

eAppendix 2. Family and Social Support Items

(1) 'How often in contact with family members in Australia?' (1= Nearly every day, 3–4 days a week, 1–2 days a week, 3=1–3 days a month, 5=Less than once a month, 6=Never, 7=No family); (2) 'How often in contact with friends in Australia?' (1=Nearly every day, 3–4 days a week, 1–2 days a week, 3=1–3 days a month, 5=Less than once a month, 6=Never, 7=No family); (3) 'Number of family members can rely on for serious problems?' (1=0 family members, 2=1–2 family members, 3=3–4 family members, 4=5 or more family members); (4) 'Number of family members can confide in for serious problems?' (1=0 family members, 2=1–2 family members, 3=3–4 family members, 4=5 or more family members); (5) 'Number of friends can rely on for serious problems?' (1=0 friends, 2=1–2 friends, 3=3–4 friends, 4=5 or more friends); (6) 'Number of friends can confide in for serious problems?' (1=0 friends, 2=1–2 friends, 3=3–4 friends, 4=5 or more friends).

© 2019 Rees SJ et al. *JAMA Network Open.*

eTable 1. Prevalence of Predictors Used in Logistic Regression Analysis by Women Born in Host Nation, Women Who Identified as Refugees, and Women From Other Conflict-Affected Backgrounds Results of Quality Assessment per Study

Predictors	Host-nation Born Women: No. (%)	Self-identifying Refugee Women: No. (%)	Conflict-affected Background Women: No. (%)	[k] p-values: Self-identifying Refugee Women vs. Host-nation Born Women	[k] p-values: Self-identifying Refugee Women vs. Conflict-affected Background Women
All	650 (100.0)	289(100.0)	396 (100.0)		
Employment status					
Employed	383 (58.1)	71 (24.6)	127 (32.1)	<0.001	0.032
Unemployed and others	267 (41.1)	218 (75.4)	269 (67.9)	<0.001	0.032
General TE counts					
None	344 (52.9)	108 (37.4)	228 (57.6)	<0.001	<0.001
One TE	182 (28.0)	95 (32.9)	117 (29.5)	0.131	0.352
Two to three TEs	103 (15.8)	67 (23.2)	45 (11.4)	0.006	<0.001
Four or more TEs	21 (3.2)	19 (6.6)	6 (1.5)	0.019	<0.001
Refugee-specific TE counts					
None	544 (83.7)	45 (15.6)	235 (59.3)	<0.001	<0.001
One TE	86 (13.2)	147 (50.9)	114 (28.8)	<0.001	<0.001
Two or more TEs	20 (3.1)	97 (33.6)	47 (11.9)	<0.001	<0.001
Intimate Partner Violence (IPV)					
No IPV and low respect	482 (74.2)	141 (48.8)	240 (60.6)	<0.001	0.002
Severe psychological IPV	133(20.5)	124 (42.9)	135 (34.1)	<0.001	0.018
Physical IPV	35 (5.4)	24 (8.3)	21 (5.3)	0.089	0.116
Index: Number of friends/family members who can be relied upon for serious problems					
Five or more members	297 (45.7)	36 (12.5)	84 (21.2)	<0.001	0.003
Three to four members	245 (37.7)	125 (43.3)	148 (37.4)	0.107	0.121
Two or fewer members	108 (16.6)	128 (44.3)	164 (41.4)	<0.001	0.453
Number of finance-related stress					
None	498 (76.6)	154 (53.3)	273 (68.9)	<0.001	<0.001
One to two stress	111 (17.1)	70 (24.2)	76 (19.2)	0.010	0.111
Three or more stress	41 (6.3)	65 (22.5)	47 (11.9)	<0.001	<0.001

[k] To compare the significant differences between two groups we used Z-test for two independent sample proportion.

© 2019 Rees SJ et al. *JAMA Network Open.*

eTable 2. Adjusted Odds Ratios (OR) With 95% CIs From Multiple Logistic Regression Analysis for Combined Sample of Women Born in Host Nation and All Women from Conflict-Affected Countries (n = 1335) Controlling for TE counts

	Model 1a [j]		Model 1b [j]	
Significant predictors [g]	Adjusted OR (95% CI)	p value for OR	Adjusted OR (95% CI)	p value for OR
Strictly defined refugee status				
Self-identifying Refugee Women	1.25 (0.84-1.87)	.263	1.63 (1.12-2.38)	.010
Other Conflict-affected Background Women	1.02 (0.70-1.48)	.914	1.03 (0.71-1.48)	.606
Host-nation Born Women (reference category)	1[Reference]		1[Reference]	
Employment status				
Employed (reference category)	1[Reference]		1[Reference]	
Unemployed	1.44 (1.02-2.02)	.039	1.48 (1.06-2.08)	.021
[I] General and Refugee TEs				
One or None (reference category)	1[Reference]			
Two TEs	1.99 (1.34-2.96)	.001	-	
Three TEs	2.22 (1.41-3.53)	<.001		
Three or more TEs	2.45 (1.56-3.86)	<.001		
IPV category				
No (reference category)	1[Reference]		1[Reference]	
Severe Psychological IPV	1.58 (1.15-2.19)	.005	1.63 (1.19-2.24)	.002
Physical IPV	4.72 (2.82-7.92)	<.001	4.85 (2.93-8.07)	<.001
Number of friends/family members can rely for serious problems				
Five or more members (reference category)	1[Reference]		1[Reference]	
Three to four members	1.35 (0.89–2.02)	.155	1.33 (0.89–1.99)	.164
Two or fewer members	1.75 (1.14-2.69)	.010	1.76 (1.15-2.68)	.009
Number of finance related stress				
None (reference category)	1[Reference]		1[Reference]	
One to two	1.85 (1.29-2.63)	.001	2.00 (1.41-2.83)	<.001
Three or more	2.08 (1.36-3.19)	.001	2.48 (1.65-3.74)	<.001

Abbreviation: MDD, Major Depressive Disorder; OR, odds ratio; TEs, Traumatic Events; IPV: Intimate Partner Violence.

[g] All the predictors included in multiple logistic regression model was found statistically significant (p<0.05) in stepwise multiple logistic regression analysis for Host-nation Born Women and All Women from Conflict-affected Countries respectively.

Significant predictors of depression (MDD) found in bivariate analysis for Host-nation Born Women (marital status, family composition, employment status, general TE counts, IPV, availability of friend/family members can rely for serious problems and number of finance-related stress) and All Women from Conflict-affected Countries (general predictors: marital status, employment status, general TE counts, IPV, availability of friends/ family members who can be relied upon for serious problems and number of finance-related stress) were included in separate stepwise multiple logistic regression analysis for Host-nation Born Women and All Women from Conflict-affected Countries respectively. In addition to these significant predictors, strictly defined refugee status (Self-identifying Refugee Women, Other Conflict-affected Background Women and Host-nation Born Women) also included in the multiple logistic regression model to explore the impact of being ever having been a refugee on MDD adjusting for all other significant predictors in the model.

[h] The outcome variable MDD for multiple logistic regression model was coded as: 1=depressed and 0=not depressed.

[i] Total TE counts constructed by adding General TE counts and TE counts generally but not exclusively related to refugees.

© 2019 Rees SJ et al. *JAMA Network Open.*

[j] Model 1a Included total TE counts and Model 1b excluded total TE counts.

Reference category: used as reference category in logistic regression analysis and OR for reference category is ‘1’.

© 2019 Rees SJ et al. *JAMA Network Open.*
